# Emerging recombination of the C2 sub-genotype of HFMD-associated CV-A4 is persistently and extensively circulating in China

**DOI:** 10.1038/s41598-019-49859-7

**Published:** 2019-09-20

**Authors:** Tianjiao Ji, Yue Guo, Likun Lv, Jianxing Wang, Yong Shi, Qiuli Yu, Fan Zhang, Wenbin Tong, Jiangtao Ma, Hanri Zeng, Hua Zhao, Yong Zhang, Taoli Han, Yang Song, Dongmei Yan, Qian Yang, Shuangli Zhu, Yan Zhang, Wenbo Xu

**Affiliations:** 1 NHC Key Laboratory of Medical Virology and Viral Diseases (National Institute for Viral Disease Control and Prevention, Chinese Center for Disease Control and Prevention), Beijing, People’s Republic of China; 2 Tianjin Municipal Center for Disease Control and Prevention, Tianjin Municipal, People’s Republic of China; 3 Shandong Center for Disease Control and Prevention, Shandong Province, People’s Republic of China; 4 Jiangxi Center for Disease Control and Prevention, Nanchang, Jiangxi Province People’s Republic of China; 5 Hebei Center for Disease Control and Prevention, Shijiazhuang, Hebei Province People’s Republic of China; 6 Hunan Center for Disease Control and Prevention, Changsha, Hunan Province People’s Republic of China; 7 Sichuan Center for Disease Control and Prevention, Chengdu, Sichuan Province People’s Republic of China; 8 Ningxia Center for Disease Control and Prevention, Yinchuan, Ningxia Province People’s Republic of China; 9 Guangdong Center for Disease Control and Prevention, Guangzhou, Guangdong Province People’s Republic of China; 10 Chongqing Center for Disease Control and Prevention, Chongqing Municipal, People’s Republic of China

**Keywords:** Non-coding RNAs, Infectious diseases

## Abstract

Sporadic outbreaks caused by coxsackievirus A4 (CV-A4) have been reported worldwide. To further elucidate the detailed genetic characteristics and evolutionary recombination events of CV-A4, virus samples from nationwide hand, foot and mouth disease (HFMD) surveillance, encompassing 27 out of the 31 provinces in China, were investigated. Comprehensive and systematic phylogenetic analyses were performed by using 29 complete genomes, 142 complete CV-A4 VP1 sequences. Four genotypes (A, B, C and D) and five sub-genotypes (C1-C5) were re-identified based on the complete VP1 sequences. C2 is the predominant sub-genotype of CV-A4 associated with HFMD and has evolved into 3 clusters. Cluster 1 is a major cluster that has been persistently and extensively circulating in China since 2006 and has been associated with all severe cases. All the sequences showed high homology with the CV-A4 prototype in the P1 region, while higher identities with CV-A5, CV-14 and CV-16 in the P2 and P3 regions. Recombination analysis revealed that C2 had two specific genetic recombination patterns with other EV-A prototypes in the 5′-UTR and 3D region compared with C5. These recombination patterns might be associated with the increased transmissibility of C2 viruses, which were obtained due to their persistent and extensive circulation in populations.

## Introduction

Hand, foot and mouth disease (HFMD), which is caused by multiple types of enteroviruses (EV), is a common childhood viral infection that is typically mild and self-limiting and often affects children under five years of age^1–3^. Enterovirus A71 (EV-A71)^4–7^ and coxsackievirus A16 (CV-A16)^8–10^ are the common aetiological agents involved in HFMD. However, other EVs of none EV-A71 and CV-A16 have often been associated with HFMD in mainland China since 2013^11–14^. Coxsackievirus A4 (CV-A4) is a member of the enterovirus A (EV-A) species. Similar to EV infections, most CV-A4 infections are usually asymptomatic, but they also lead to a wide spectrum of clinical diseases ranging from mild to severe cases or even fatal cases, such as hand, foot and mouth disease (HFMD^7,15,16^), herpangina (HA)^17^, acute flaccid paralysis (AFP)^18^, myocarditis^19^ and severe central nervous system symptoms^20^. Several epidemics caused by CV-A4 in many regions indicate that CV-A4 is an important pathogen and co-circulates with other EVs^15,16^. Two HFMD outbreaks occurred in Taiwan in 2004 and 2006, both of which were caused by CV-A4^16^. After these outbreaks, CV-A4 raised concerns again when it began to co-circulate with CV-A16 in 2010. A febrile disease outbreak with a limited scope occurred in a nursery school in Beijing on June 2011, and CV-A4 was confirmed to be the primary pathogen^21^. CV-A4 was also identified as the pathogen in two HA outbreaks in Shenzhen in 2012 and 2014^22^.

EV belongs to the Picornaviridae family, and its genome (approximately 7,450 nucleotides) is positive-sense, single-stranded, and contains a long open reading frame (ORF) flanked by a 5′-untranslated region (UTR) and a 3′-UTR^23^. The ORF is composed of 3 protein precursors: P1, P2, and P3. The P1 protein encodes the 4 structural polypeptides VP1–VP4. VP1 plays a critical role in mediating the function of the binding receptor, and complete VP1 has been widely used to identify EV serotypes because its coding region contains many important neutralizing antigenic sites^24^. P2 and P3 are precursors of the non-structural proteins 2A–2C and 3A–3D, respectively.

Several phylogenetic analyses based on the partial VP1 sequences of worldwide CV-A4 samples were conducted^16,18^, however, further analyses of the genetic characteristics and recombination of CV-A4 were limited because of the lack of full-length VP1 and complete genome sequences. In this study, we performed a nationwide analysis using 142 complete VP1 sequences of CV-A4 samples from 1996 and 2017, together with 13 complete genome sequences determined in this study to provide a comprehensive molecular characterization and recombination analysis of CV-A4.

## Results

### CV-A4 isolates associated with HFMD cases in China from 2008 to 2017

From 2008 to 2017, a total of 18242 EV-associated HFMD samples were collected. A total of 4212 samples were determined to be EVs other than EV-A71 and CV-A16. Among these non-EV-A71 and non-CVA16 EVs, there were 288 CV-A4 viruses, which represented the third most abundant virus, behind CV-A6 (1866) and CV-A10 (1002) (Supplementary Table S1) in mainland China.

The 288 CV-A4 viruses obtained in this study were collected from 27 out of the 31 provinces in mainland China (Supplementary Table S1). The data showed that CV-A4 has been extensively spread every year since 2014, as CV-A4 had a limited distribution in regions and provinces and fewer cases before 2014.

The median age of these patients was 2.00 years (range 0.4–7 years), and most patients (176/227, 77.53%) were under 3 years old. More than 83% (189/227) of the HFMD cases occurred in April and July, during spring and summer in China. Patients with neurological complications or cardiopulmonary complications were diagnosed as severe cases, according to the National Guideline for Diagnosis and Management of HFMD cases issued by National Health Commission of China in 2018^25^. In this study, 17 cases were identified as severe cases, and all of the patients with severe HFMD were less than 4 years old.

### Four CV-A4 genotypes were assigned based on the complete VP1 sequence

**Figure 1:**
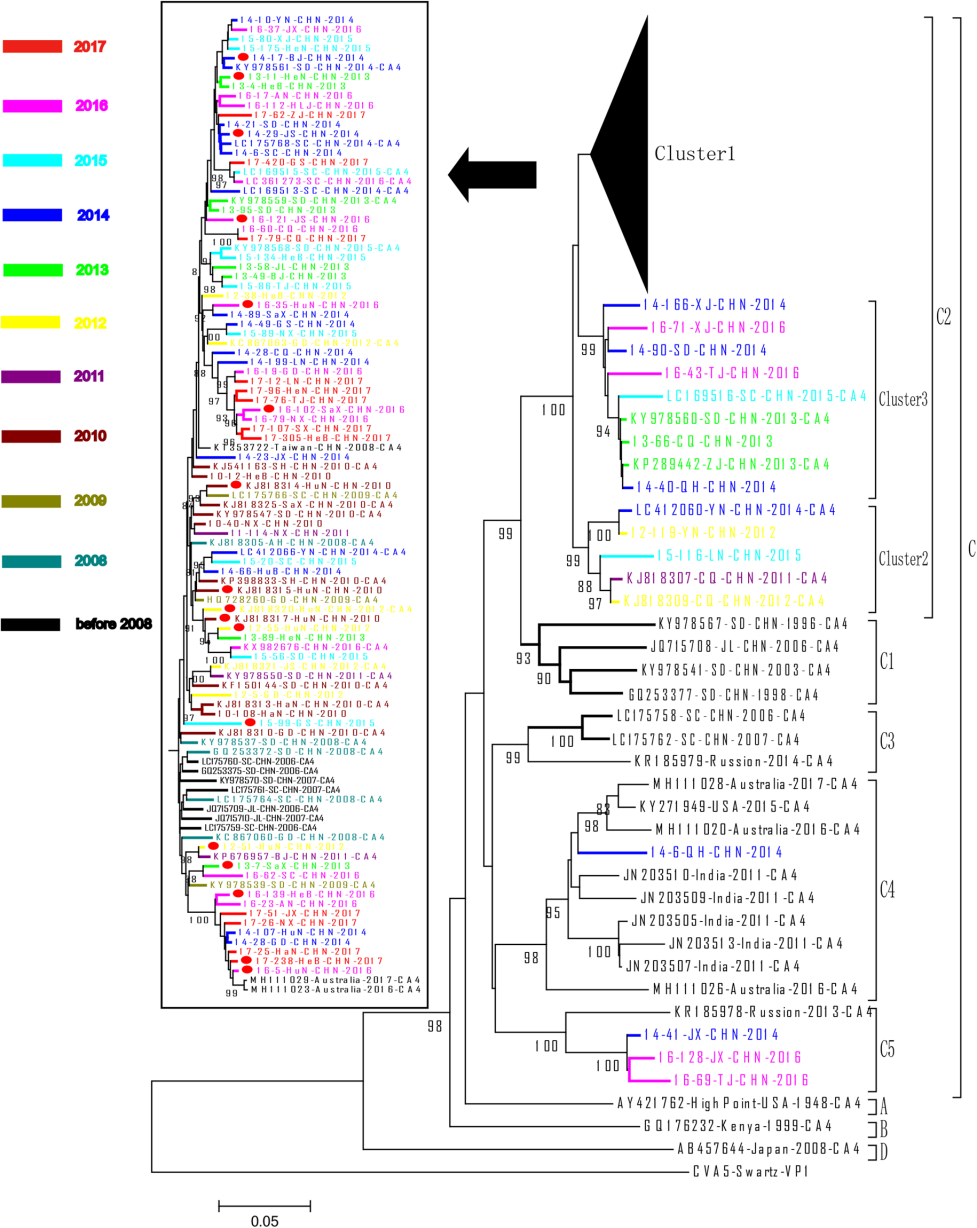
A phylogenetic dendrogram *(neighbor-joining method)* based on the 915 nt sequences of 142 representative CV-A4 isolates collected from 1948–2017. The strains isolated in different years are represented by different colours according to the legend. A solid circle indicates severe cases. The sequences downloaded from GenBank are listed in Supplementary Table S3. The prototype CV-A5 strain (Swartz) served as an out-group reference.

A total of 166 VP1 sequences of CV-A4 were available from GenBank until December 31^st^, 2018. These sequences were isolated from samples in 6 countries from 1948 to 2016 and are associated with different populations, including cases of HFMD, AFP, febrile illness and undefined disease as well as healthy people. Representative sequences of all the locations/countries/provinces and time ranges were selected to be included in the phylogenetic tree. Sequences with high homology (identical or more than 99% nucleotide homology) or with significant errors in sequences were not included in this study. In total, *a phylogenetic dendrogram was constructed with 142 CV-A4 VP1 sequences using the neighbour-joining method (*Fig. 1*) and Maximum Likelihood method* (Supplementary Fig. S1*) in MEGA 5*.*0* and included 74 of the 288 VP1 sequences from this study and 68 of the 166 sequences from GenBank (Supplementary Table S2).

According to published genotyping criteria that suggest a difference of at least 15% among complete VP1 nucleotide sequences should be used to distinguish genotypes^22,23^, the phylogenetic tree of VP1 clearly indicated that all CV-A4 strains are grouped into 4 distinct genotypes, which were designated as A, B, C, and D. The nucleotide divergence among the A, B, C and D genotypes ranged from 15.19%~25.14%, 15.19%~26.45% and 24.84%~26.45%, respectively (Table 1). It is noteworthy that the maximum nucleotide divergence between genotype D (AB457644-Japan-2008-CA4) and other genotypes reached 26.45%, which was beyond the threshold for a new genotype. Additionally, the nucleotide divergence between genotype D and the other EV-A serotype prototype sequences were even larger (36.7%~44.8%). Therefore, this Japanese strain (AB457644-Japan-2008-CA4) was still classified as CV-A4. Genotype C could be clustered into 5 sub-genotypes, C1-C5. The mean nucleotide difference among the C1-C5 sub-genotypes was 12.8%~14.6%.

**Table 1:** Estimates of evolutionary divergence over sequence pairs between genotypes/sub-genotypes.

Genotype	Evolutionary Divergence							
	A	B	D	C1	C2	C3	C4	C5
A	*/*	*6*.*55%*	*17*.*59%*	*8*.*79%*	*8*.*17%*	*8*.*74%*	*9*.*07%*	*9*.*05%*
B	15.19%	*/*	*16*.*90%*	*9*.*74%*	*9*.*14%*	*10*.*57%*	*9*.*24%*	*9*.*14%*
D	25.14%	26.45%	*/*	*16*.*12%*	*16*.*50%*	*17*.*70%*	*17*.*07%*	*16*.*72%*
C1	16.28%	17.60%	24.92%	***8***.***69%***	*5*.*78%*	*7*.*01%*	*7*.*77%*	*7*.*18%*
C2	15.21%	17.58%	25.50%	11.54%	***5***.***42%***	*7*.*91%*	*8*.*48%*	*7*.*61%*
C3	14.79%	17.74%	26.01%	13.88%	13.82%	***7***.***07%***	*6*.*92%*	*7*.*07%*
C4	15.65%	17.99%	24.19%	14.40%	14.88%	12.96%	***7***.***07%***	*6*.*87%*
C5	15.41%	17.79%	24.84%	15.22%	15.40%	14.57%	14.87%	***6***.***12%***

Note: The lower-left data are nucleotide diversity. The upper-right data in italic are deduced amino acid sequence diversity. The mean nucleotide diversities within genotypes/sub-genotypes (this study) are marked with an underline in bold.

Genotypes A, B and D included prototype CV-A4 strains (High Point strain) isolated from North Carolina in the United States of America in 1948, Kenya in East Africa in 1999, and Japan in 2008, respectively. Genotype C was composed of viruses detected in China, Russia, India, Australia and the USA, and these viruses were associated with several diseases, including HFMD, AFP, febrile illness and enterovirus infection, from 1996–2017. The genotype C strains, especially the sub-genotypes C1~C3 and C5, were mainly composed of Chinese sequences except for one strain isolated from Russia in 2013 and two strains isolated from Australia in 2016 and 2017. The sub-genotype C4 was mainly composed of non-China strains with one strain from Qinghai, China (14-6-QH-CHN-2014). Most of the other Chinese strains (116/126, 92.06%) belonged to C2 and encompassed 7 geographic regions and 26 of the 31 provinces in China during 2006–2016, including one strain from Taiwan, China in 2008. Based on the continuous and extensive surveillance of HFMD in China, the transmission of C1 and C3 might have been interrupted since 2008.

### Persistent circulation of sub-genotype C2 in mainland China from 1996 to 2017

All the CV-A4 strains isolated since 2008 circulating in China were clustered in the same group as the sub-genotype C2, C4 and C5 isolates, and most of these (96.46%, 109/113) isolates belonged to C2. The strain 14-6/QH/CHN/2014 was the sole Chinese member of C4, and three strains from Jiangxi and Tianjin (14–41/JX/CHN/2014, 16-128/JX/CHN/2016 and 16–69/TJ/CNH/2016) were assigned to C5. The isolates of the C4 sub-genotype were geographically limited to Northwest China, and C5 isolates were only detected in northern China and eastern China.

**Figure 2:**
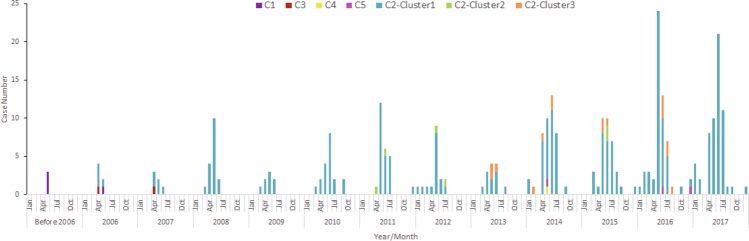
Yearly distribution of the CV-A4 sub-genotypes in China from 1996 to 2016. Different sub-genotypes are coloured according to the legend.

Sub-genotype C2 was found beginning in 2006 and became increasingly prevalent each year, while sub-genotypes C3/C1 were found only before 2008 and C4/C5 have been detected with limited numbers since 2014 (Fig. 2). All the other Chinese viruses together with the viruses circulating in other countries grouped in C2 could be categorized into 3 clusters: C2-cluster1, C2-cluster2, and C2-cluster3. The group mean distance among these 3 clusters ranged from 6.47% to 6.69%. No differences were found among these three clusters in temporal and geographic distribution. However, C2-cluster 1 was the largest branch with the most extensive geographic distribution, found in 26 of the 31 provinces, and all 17 severe cases were included in this cluster.

### Complete genome sequence analysis of CV-A4

The full-length genomes of the 13 representative CV-A4 strains were sequenced, including 11 representative C2 strains and 2 representative C5 strains. Five C2 strains were isolated from severe cases, while six C2 and two C5 strains were isolated from mild cases. No deletions or insertions were observed in the P1, P2, and P3 genomic regions of any CV-A4 strains, and all strains had an ORF length of 6606 nucleotides, encoding a polypeptide of 2202 amino acids.

For further genomic analysis, 29 complete genomic CV-A4 sequences were used (Supplementary Table S3), including 13 obtained from this study, one prototype, and 15 retrieved from GenBank (11 strains from China in 2009–2013, 3 strains from Australia in 2016–2017, and 1 strain from USA in 2015). From the phylogenetic analysis, these 29 isolates were grouped into genotype A (1), sub-genotype C2 (23), C4 (3) and C5 (2) based on the complete VP1 region, and most of the C2 sequences were from China (except for MH111029-Australia-2017-CA4). Nucleotide substitutions among different CV-A4 strains were scattered throughout the genome. All the CV-A4 full-length sequences had 83.6–97.7% shared nucleotide sequence identity and 96.5%–99.4% shared amino acid sequence identity in the coding region except for the prototype High Point. Two representative C2 sequences (14-17/BJ/CHN/2014 and 12-119/YN/CHN/2012), one representative C5 sequence (16-128/JX/CHN/2016) and one representative C4 strain from USA were selected to perform the phylogenetic comparisons of these 4 strains with the CV-A prototype strains (High Point) and the prototype of other EV-A (Table 2). We found that, in the capsid region, the nucleotide sequences of the 4 representative CV-A4 isolates were closer to the CV-A4 prototype strain (83.0%–87.0%) than to the other CV-A prototype strains (51.2%–72.9%), while in the P2 and P3 regions, these four sequences, especially the two C2 strains, showed more homology with the other CV-A prototype strains than with the High Point strain, which indicated the occurrence of recombination events in a non-capsid region. Further comparative analysis of the 29 genomic CV-A4 sequences and the EV-A prototype strains could also support this hypothesis (Supplementary Table S4): the P1 and the mature VP1-VP4 regions were 84.0%–86.1%, 84.4%–85.6%, 94.1%–99.2%, 83.0%–87.5% and 95.6–100% homology, respectively, with CV-A4. However, in the P2 and P3 regions (except for the 2B and 3 C regions), the sequences showed significantly higher similarity with CV-14 and CV-16, ranging from 77.2%–90.0% and 76.7%–87.7%, respectively, than with CV-A4 and other CV-A prototypes, which had 77.1%–86.7% and 56.0%–83.3% sequence similarity, respectively.

**Table 2:** Nucleotide and amino acid identities of 4 CV-A4 sequences and the EV-A prototype strains.

Region	Nucleotide identity (%)							
	14-17/BJ/CHN/2014(C2/severe case)		12-119/YN/CHN/2012(C2/mild case)		16-128/JX/CHN/2016(C5/mild case)		KY271949-USA-2015(C4)	
	High Point	Other EV-A prototype	High Point	Other EV-A prototype	High Point	Other EV-A prototype	High Point	Other EV-A prototype
5′ UTR	87.6	70.0–87.6	88.3	69.4–85.0	88.3	68.2–86.6	85.1	62.7–80.1
VP4	86.4	65.2–71.4	85.0	64.7–72.4	84.5	64.2–70.5	84.5	65.7–72.9
VP2	85.6	62.8–70.3	85.6	63.5–70.8	85.4	64.7–70.7	85.1	63.4–70.7
VP3	87.0	62.5–71.6	85.1	63.0–71.6	85.4	63.7–71.3	83.0	63.0–71.2
VP1	85.0	51.2–60.0	85.6	52.3–62.7	84.6	52.4–60.3	83.9	53.1–61.2
2 A	81.7	66.2–83.3	82.0	65.5–80.8	77.7	66.4–83.1	81.1	65.5–81.7
2B	83.1	67.3–84.5	80.4	66.3–83.8	84.1	67.0–84.8	78.7	68.6–84.5
2 C	82.9	72.3–85.6	85.1	72.6–86.4	82.7	73.4–83.9	83.3	73.9–83.6
3 A	82.5	67.4–85.9	83.3	69.3–81.1	83.8	67.0–83.7	83.3	68.6–86.4
3B	84.8	60.6–87.8	87.8	60.6–89.3	81.8	59.0–77.2	83.3	54.5–84.8
3 C	82.8	72.3–83.6	82.8	73.4–83.4	86.7	71.2–83.9	81.6	70.1–81.0
3D	83.1	71.9–83.2	84.4	72.5–84.8	84.1	73.1–84.3	84.7	71.8–83.8
3′UTR	91.3	29.7–90.1	92.5	29.7–90.1	92.5	28.7–92.5	86.4	28.1–83.9

**Figure 3:**
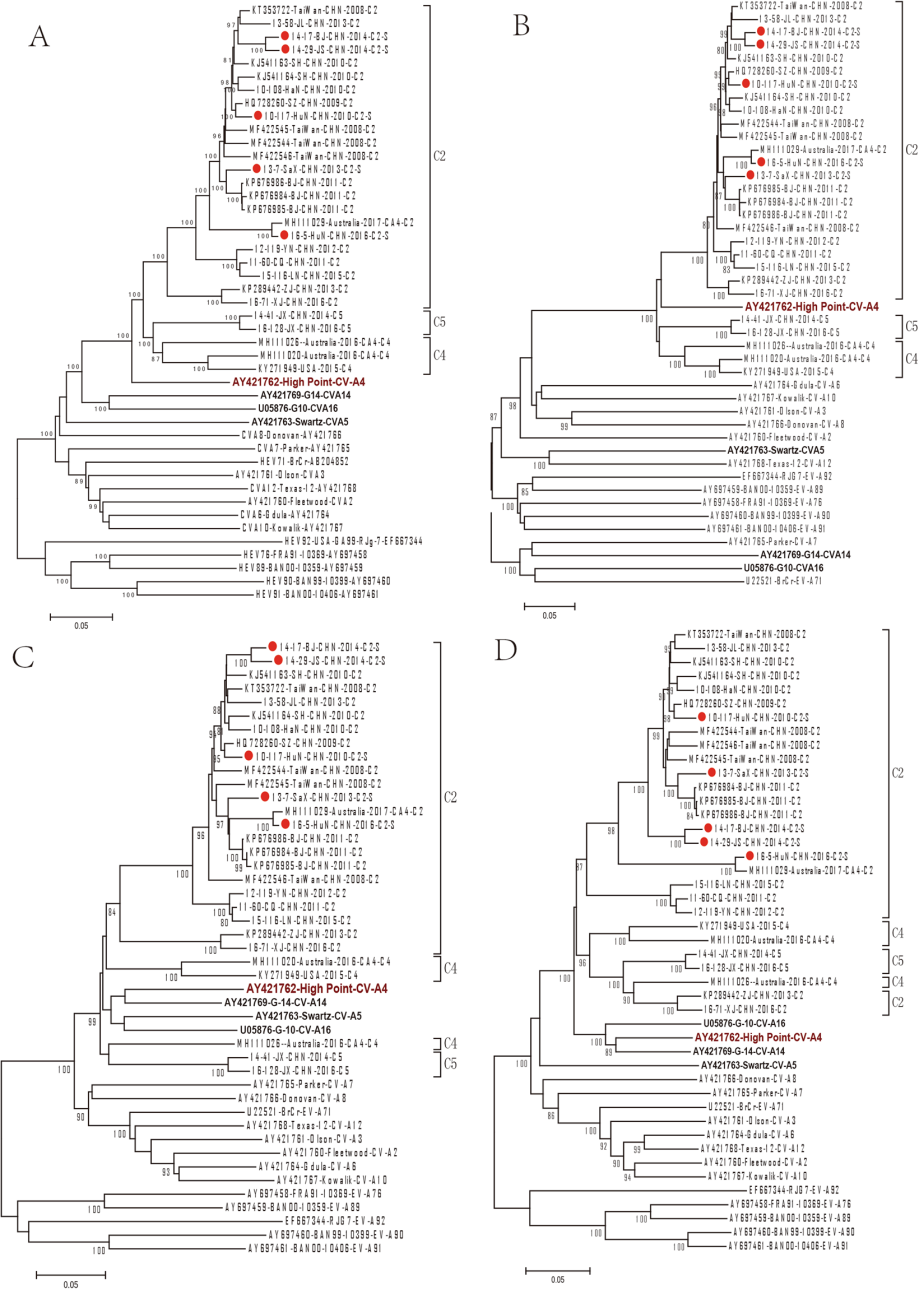
*The neighbor-joining trees* constructed from CV-A4 strains and EV-A prototypes based on the whole genome (**A**) and the P1 (**B**), P2 (**C**), P3 (**D**) structural protein coding regions. Hollow circles indicate strains isolated from mild cases in this study, and rhombus indicates strains isolated from severe cases in this study.

Phylogenetic dendrograms based on the whole genome, protein encoding regions, and each individual gene were performed with MEGA 5.0 software, *by using both neighbour-joining and Maximum Likelihood method*, *as shown in* Fig. 3 *and Supplementary* Figs S2–S5. Based on the whole genome, P1 and its encoded proteins, all of the 29 sequences displayed a close phylogenetic relationship with the CV-A4 prototype (Figs 3A,B and S1), and C2 showed more consistent nucleotide identities with the CV-A4 prototype than C4 and C5 (Table 2). Based on P2 and P3 (Fig. 3C,D), we found that all 29 sequences clustered within a clade, including the CV-A4, CV-A5, CV-A14 and CV-A16 prototypes.

Moreover, different topology structures were found in the C2 and C4 strains in the P2 and P3 regions. All the C2 sequences were grouped into one cluster in P2 but were divided into 2 branches in the P3 regions, with two strains (KP289442/ZJ/CHN/2013 and 16-71/XJ/CHN/2016) clustered with the C4 and C5 sequences (Fig. 3D). According to the p-distance analysis, the C2 sequences showed higher homology to CV-A14, except for the two strains mentioned above, which had a closer relationship to the CV-A4 prototype. The C4 isolates were divided into 2 branches in both the P2 region, which showed close homology with CV-A16, and the P3 region, showed close homology with the CV-A4 prototype.

In addition, we found more recombination patterns based on detailed phylogenetic analysis of each encoded gene and noncoding region (Supplementary Figs S3–S5). According to the dendrogram based on the 5′-UTR region, we found that the C2 and C5 sequences were clustered with the CV-A6 prototype, while the C4 sequences were located in the same clade with the prototypes of CV-A4, CV-A14 and CV-A16. The phylogenetic tree of each mature P3 protein was more complicated. In the 3A region, five C2 strains were clustered with C5 and showed a close phylogenetic relationship with CV-A5. However, in the 3D region, the C2 strains could be divided into 3 branches, and two C2 strains, including one strain isolated from a severe case (16-5-HuN-CHN-2016), were separated from the other CV-A4 strains.

In summary, all these data indicate different topology of the phylogenetic dendrograms between the P1 and P2-P3 regions, indicating the potential recombination of these circulating CV-A4 strains with other EV-A serotypes in genomic regions encoding non-structural proteins.

### Mutations and recombination with other EVs might change the transmissibility of sub-genotype C2

**Figure 4:**
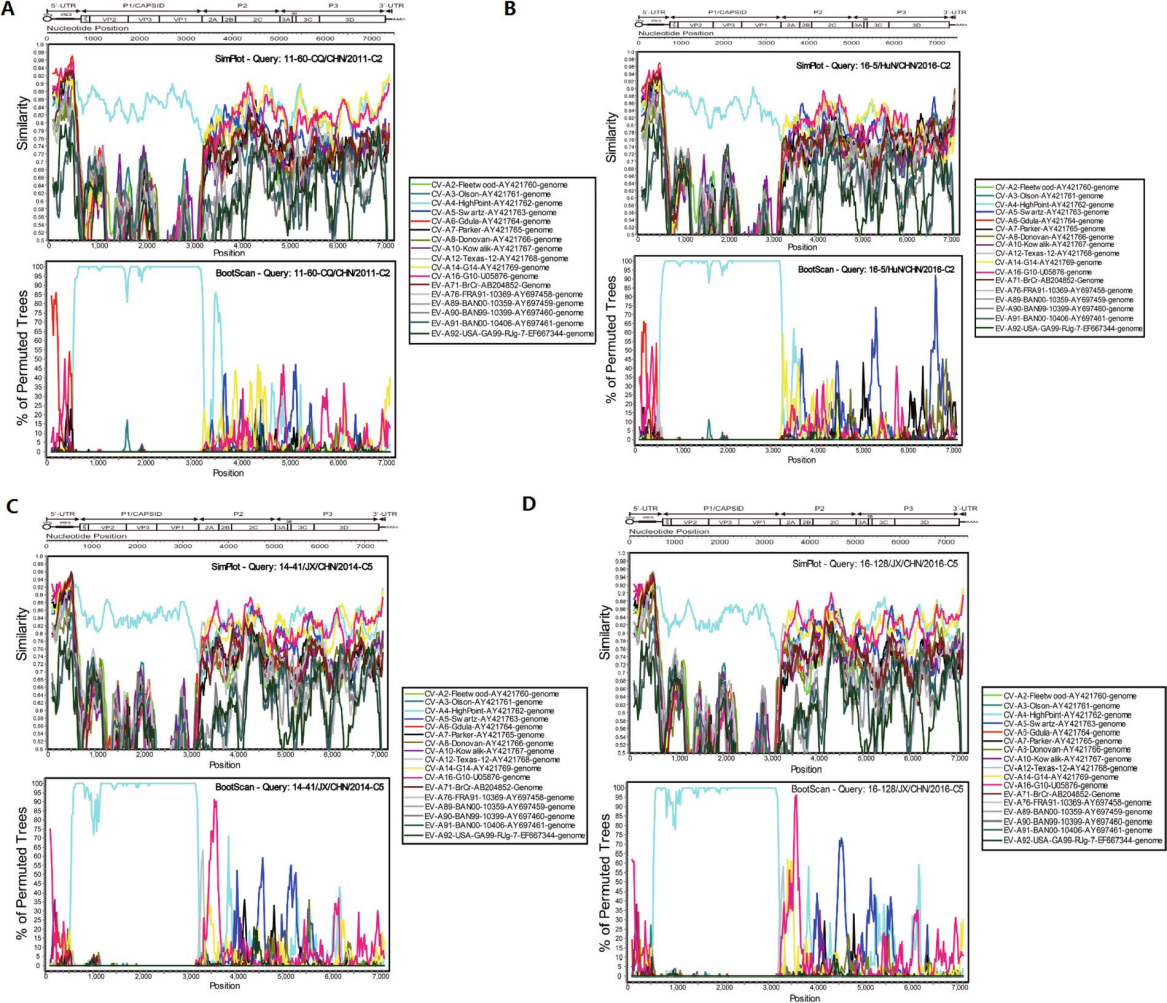
Similarity plot and bootscan analysis of representative CV-A4 strains of HFMD circulating in China with the EV-A prototype strains. A sliding window of 500 nucleotides moving in 20 nucleotide increments was used in this analysis. (**A**) Mild case of C2, 11–60-CQ-CHN-2011. (**B**) Severe case of C2, 16–5-HuN-CHN-2016. (**C**) C5, 14–41-JX-CHN-2014. (**D**) C5, 16–128-JX-CHN-2016.

To further clarify the possible recombination of CV-A4 strains circulating in China, similarity plot and bootscan analyses were performed by using the EV-A prototype strains as reference sequences (Fig. 4 and Supplementary Figs S6–S7). All the C2 strains showed high similarity with the CV-A4 prototype strain High Point in the P1 region. However, in the 5′ UTR, C2 strains exhibited high similarity with CV-A6, CV-A14 and CV-A16, in addition to the CV-A4 prototype, and bootscan analysis also confirmed that a potential recombination of approximately 250 bp was donated from CV-A6. Similarly, in the P2 and P3 regions, as indicated in the similarity plot analysis, all the C2 strains showed high similarity with CV-A4, CV-A5, CV-A14 and CV-A16. It is interesting that, according to the bootscan analysis, all the C2 strains isolated from 5 severe cases showed recombination with CV-A5 in the 3C and 3D regions, while four of the six C2 strains isolated from mild cases did not show this recombination. Similarly, all the C5 strains from mild cases were analysed with the same method, and only a small section in the 2A region showed recombination with CV-A16.

## Discussion

As a member of EV-A, CV-A4 has circulated in the world for many years since the prototype strain High Point was isolated in 1948^23^. Since 2003, sporadic cases and outbreaks of CV-A4 have been reported, and CV-A4 was also found to be circulating with increasing frequency in several countries worldwide, including China (mainland China^26^, Taiwan in China^16,27^), Singapore^7^, Thailand^28^ and Australia^29^. In China, CV-A4 has gradually emerged as an important factor in EV infections since 2008, when HFMD was considered the 38th legally notifiable disease in China’s National Notifiable Disease Reporting and Surveillance System. Additionally, in this study, we found that CV-A4 has become increasingly prevalent, with increasing patients from increasing provinces impacted after 2013. This finding indicated that CV-A4, together with CV-A6 and CV-A10, is becoming a predominant serotype among the spectrum of pathogens and might be a potential common pathogen of HFMD, which is consistent with other studies^30,31^.

Several spatiotemporal studies have been conducted based on partial VP1 sequences worldwide, and CV-A4 can be divided into 2 widely divergent lineages^16,18^. Recently, Weng *et al*.^32^ conducted an analysis of CV-A4 based on the complete VP1 region sequences available through 2016, and 5 genotypes (A, B, C, D and E) were identified, which was different from our results. A total of 51 strains were used in their study, and most of them were isolated from 2008 to 2014. The different sequences included in these two studies might have caused the differences in the topological structures of dendrograms. Our study performed a comprehensive and systemic molecular epidemiologic analysis to clarify the global genotypes of CV-A4. The viruses involved in this study covered more than 20 years and used 166 complete VP1 sequences from GenBank together with 288 sequences obtained in this study. Four genotypes (A, B, C and D) were identified, and most of the strains belonged to genotype C. The other three genotypes, A, B and D, were composed of a single strain from Kenya, Japan and the prototype CV-A4 strain, respectively. Additionally, although the nucleotide diversity of the Indian sequences and the A, B and D genotypes were slightly more than 15%, which was the threshold for a new genotype, these Indian strains were clustered within the same group with the C4 sub-genotype in this study. Therefore, Indian strains still be classified into sub-genotype C4 in this study.

Genotype C can be further divided into 5 sub-genotypes (C1-C5), and C2 was the predominant sub-genotype in China. Interestingly, we found that the sub-genotypes C1 and C3 mainly circulated in 1996–2007, followed by the sub-genotype of C2 becoming prevalent in 2006–2017. Moreover, the sub-genotypes C4 and C5 were not found until 2014. According to a previous study, the ancestor of CV-A4 in China could be tracked back to 1978 and originated from Yunnan Province^18^. Therefore, we deduced that the ancestral CV-A4 initially diverged into 2 distinct lineages (C1 and C3) from 1978 to 2007. Then, after 2007, these sub-genotypes became extinct and were replaced by the C2 sub-genotype. Afterwards, the C2 sub-genotype evolved continually and diverged into 3 evolutionary clusters, including C2-cluster1, C2-cluster2 and C2-cluster3, while the C5 genotype evolved separately with 3 strains from Jiangxi and Tianjin provinces of China in 2014 and 2016. Among the 3 clusters of the C2 sub-genotype, cluster 1 was the predominant evolutionary branch, covering 26 provinces/regions/countries in 2006–2017.

Enteroviruses can exhibit rapid variation under certain evolutionary pressures. Thus, HFMD pathogens were diversified and evolved into multiple genotypes. As reported by previous studies, the estimated molecular evolution rate of CV-A4 was approximately 6.4 × 10^−3^ to 8.65 × 10^−3^ substitutions/site/year^16,18^, which is faster than the evolutionary rates of EV-A71 (4.2 × 10^−3^ to 4.6 × 10^−3^ substitutions/site/year)^6^, CV-A16 (2.49 × 10^−3^ substitutions/site/year)^10^ and CV-A6 (4.5 × 10^−3^ substitutions/site/year)^33^, and exhibited similar evolutionary rates with CV-A10 (6.2 × 10^−3^ to 6.4 × 10^−3^ substitutions/site/year)^33^. Therefore, we infer that other EVs, especially CV-A10 and CV-A4, have a high population infection rate in human populations and could be potential pathogens in large epidemics in the future.

Many studies have demonstrated that recombination and mutations are the main determinants for viral sequence variation^15,34,35^. Recombination, especially the intertypic recombination that was observed in the genomic regions encoding non-structural proteins such as enzymes and small active molecules, might lead to changes in the environmental adaptations of viruses and disease severity^36,37^. In our study, all the complete CV-A4 genomes showed high similarity to the prototype in the P1 region, which revealed that P1 was a relatively conserved region in EV. However, in non-structural regions, significant recombination with other EVs was found. As the predominant sub-genotype of CV-A4, C2 had two genetic recombination patterns with other EV-A prototypes: A recombination with CV-A6 in the 5′ UTR and a recombination with CV-A5 in the P3 region of all 5 severe cases. However, only a small section in the 2A region of C5 strains showed recombination with CV-A16. *In addition*, *we found that all the C2 strains isolated from severe cases showed recombination with CV-A5 in the 3C and 3D regions*, *while C2 strains isolated from mild cases did not show this recombination*. *Though the mechanism of recombination was not very clear*, *many studies indicate that non-structural protein was associated with viral virulence:* 5′ UTR is considered the critical region for regulating the synthesis of the viral genome and the initiation of viral protein translation^38,39^. As an RNA-dependent RNA polymerase, changes in 3D have been associated with altering the virulence of viruses^40,41^.

*Therefore, we inferred that wide distribution of C2 in mainland China in recent years might be associated with its recombination patterns, and recombination with CV-A5 in the 3C and 3D regions probably resulted in increased transmissibility and virulence, as they were associated with all severe cases*. However, continuous and extensive surveillance to include more severe cases and further genetic and animal studies to provide more evidence are needed to support this deduction.

All these findings indicated that the C2-cluster 1 viruses might have evolved with high transmissibility and virulence during the persistent and extensive circulation among populations. However, the predominant pathogens causing HFMD are variable and alternate. As there was no cross immunity among the different EV serotypes, if EV-A71 and CV-Al6 built an immune barrier by natural infection or vaccination and reached a sufficiently high level in certain populations, another serotype strain would probably become the dominant strain associated with HFMD. To elucidate additional detailed genetic characteristics and evolutionary recombination events in EV, continuous and extensive surveillance is necessary.

## Methods

### Sample collection and virus isolation

Stool, throat swabs, or nasal swabs from HFMD patients were collected according to standard protocols and were incubated in RD or HEp-2 cell lines, which were obtained from WHO Global Poliovirus Specialized Laboratory, US CDC. When cytopathic effects were observed, the isolates were harvested and shipped to the national reference laboratory for further study.

### RT-PCR amplification and determination of VP1 and whole genome sequences

A total of 288 CV-A4 isolates were obtained in this study (Supplementary Table S1), and viral RNA was extracted using a QIAamp Viral RNA Mini Kit (Qiagen, Valencia, CA, USA). The entire VP1 region of these strains was amplified with specific primers designed in this study using Primer 3.0 (Supplementary Table S5). The complete genome sequences of the 13 representative CV-A4 isolates mentioned above were determined in this study with multiple primers (Supplementary Table S5). RT-PCR was performed using the One Step PT-PCR Kit Ver. 2 (TaKaRa, # RR057A). PCR products were purified for sequencing using the QIAquick Gel Extraction Kit (Qiagen). Then, the amplicons were bi-directionally sequenced using fluorescent dideoxy terminators and an ABI PRISM 3100 Genetic Analyzer (Applied Biosystems Foster City, CA, USA).

### Phylogenetic analysis

The entire VP1 or complete genome sequences of the CV-A4 viruses were aligned with the MEGA (Version 5.03) program (Sudhir Kumar, Arizona State University, Tempe, Arizona, USA)^42^ using the reference sequences downloaded from GenBank. Phylogenetic analysis using neighbour-joining (NJ) and maximum likelihood (ML) was performed. Phylogenetic trees that represented different regions were constructed with Kimura-2 parameter evolutionary models, and the reliability of the trees were tested by 1000 bootstrap replicates. Bootstrap values greater than 80% were considered statistically significant for grouping. Estimates of evolutionary divergence between sequences were conducted in MEGA with a p-distance model.

### Recombination analysis

Alignment of the complete genome sequences was performed with the Clustal W package in the MEGA program. Potential recombination between CV-A4 and other HEV-A strains was determined with similarity plots and the bootscan method (version 3.5.1; Stuart Ray, Johns Hopkins University, Baltimore, Maryland, USA)^43^. The NJ method and Kimura 2-parameter substitution model were used. A sliding window size of 500 bp nucleotides in 20 bp nucleotide increments was used in this analysis.

### Ethics Statement

This study did not involve human participants or human experimentation. Only specimen (stool samples, throat swab samples) collected from HFMD patients for public health purposes at the urging of the Ministry of Health, P. R. of China. Written informed consent for the use of their clinical samples was obtained from the parents of the children whose samples were analyzed. This study was approved by the second session of the Ethics Review Committee of the National Institute for Viral Disease Control and Prevention (NIVDC), Chinese Center for Disease Control and Prevention, all experimental protocols were approved by NIVDC, and the methods were carried out in accordance with the approved guidelines.

## Supplementary information

supplemental figures

supplemental tables

## Data Availability

All of the complete VP1 nucleotide sequences representing different years and genotypes/clusters from this study were deposited in the GenBank database under the accession numbers MK388444-MK388504 (Supplementary Table S2), and the accession numbers for the complete genome nucleotide sequences were MK391063-MK391075 (Supplementary Table S3).
