# Supplementary material for: Emerging recombination of the C2 sub-genotype of HFMD-associated CV-A4 is persistently and extensively circulating in China
Source: Sci Rep. 2019 Sep 20;9:13668. doi: 10.1038/s41598-019-49859-7 (PMC6754396; doi:10.1038/s41598-019-49859-7)
Supplement: Supplementary file 1 — supplemental figures [file 41598_2019_49859_MOESM1_ESM.pdf]

Emerging recombination of the C2 sub-genotype of HFMD-associated CV-A4 is persistently and extensively circulating in China

Tianjiao Ji[1], Yue Guo[1], Likun Lv[2], Jianxing Wang[3], Yong Shi[4], Qiuli Yu[5], Fan Zhang[6], Wenbin Tong[7], Jiangtao Ma[8], Hanri Zeng[9], Hua Zhao[10], Yong Zhang[1],Taoli Han[1], Yang Song[1], Dongmei Yan[1], Qian Yang[1], Shuangli Zhu[1], Yan Zhang[1]* and Wenbo Xu[1]*

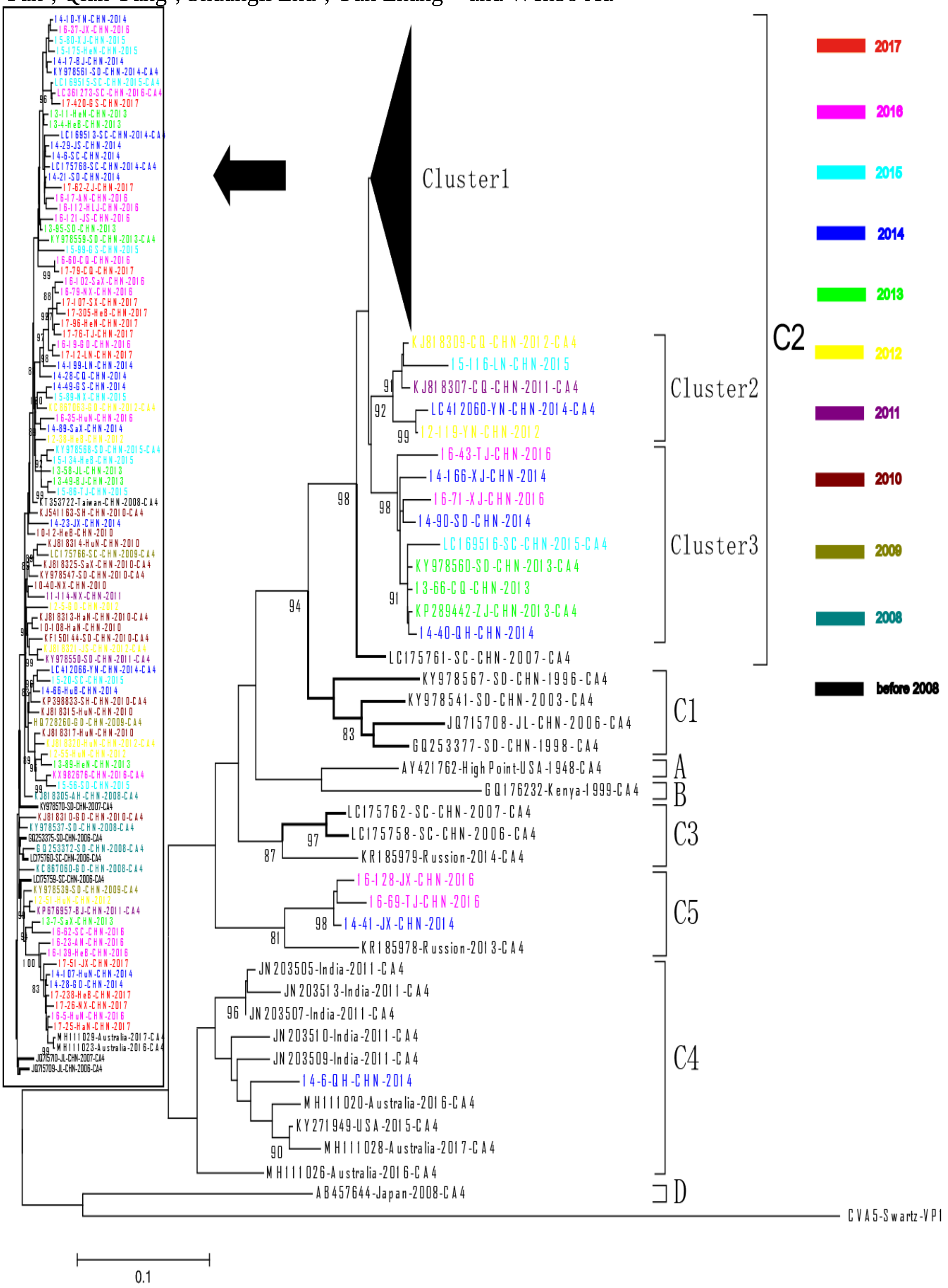

Supplementary Figures S1: A phylogenetic dendrogram (Maximum Likelihood method) based on the 915 nt sequences of 142 representative CV-A4 isolates collected from 1948-2017. The strains isolated in different years are represented by different colours according to the legend. A solid circle indicates severe cases. The sequences downloaded from GenBank are listed in Supplementary Table S3. The prototype CV-A5 strain (Swartz) served as an out-group reference.

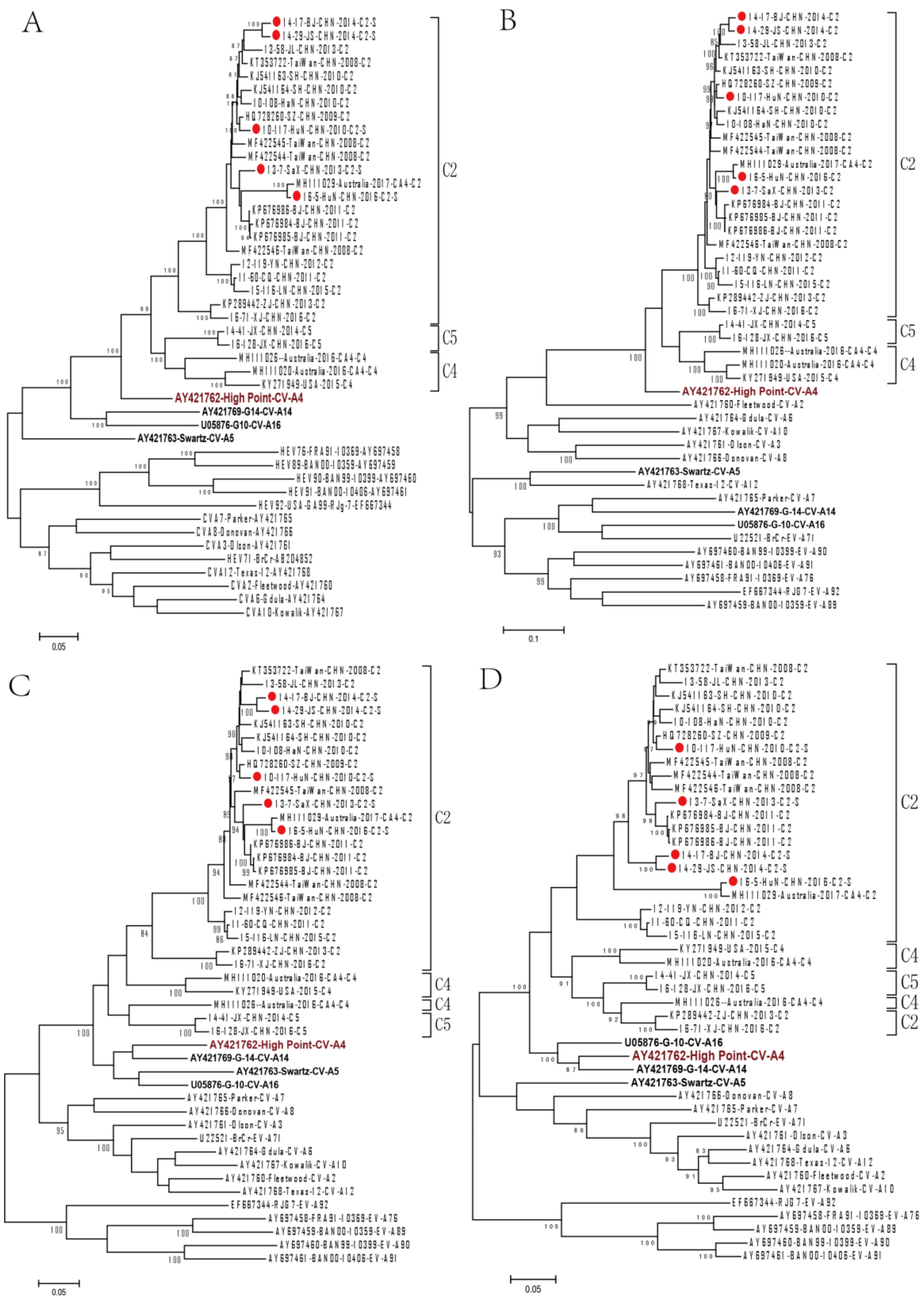

Supplementary Figures S2: The Maximum Likelihood trees constructed from CV-A4 strains and EV-A prototypes based on the whole genome (A) and the P1 (B), P2 (C), P3 (D) structural protein coding regions. Hollow circles indicate strains isolated from mild cases in this study, and rhombus indicates strains isolated from severe cases in this study

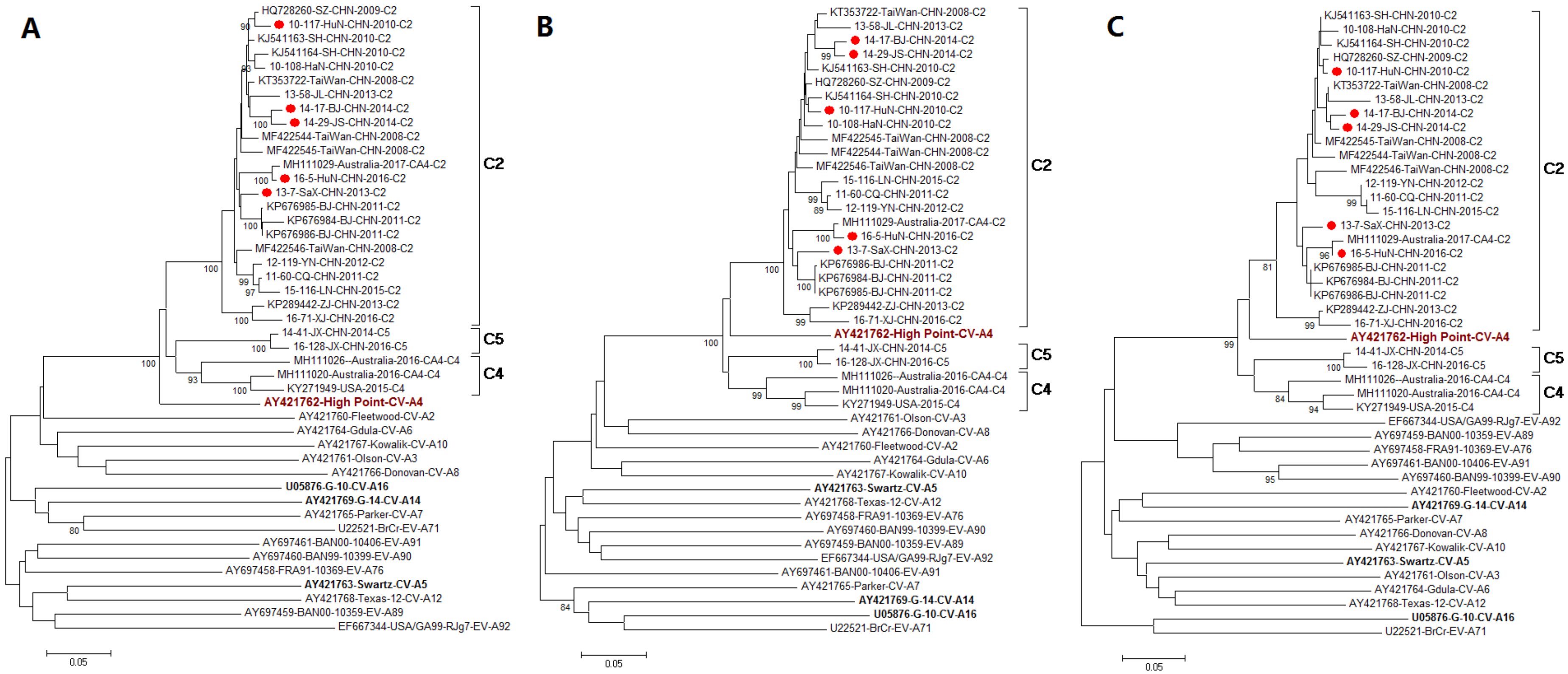

Supplementary Figures S3: Phylogenetic dendrograms showing the relationships amongst EV-A isolates using the different genomic regions. The neighbor-joining trees were constructed from alignment of the VP2 (A), VP3 (B), VP4 (C), respectively. Solid circle indicated strains isolates of severe cases in this study.

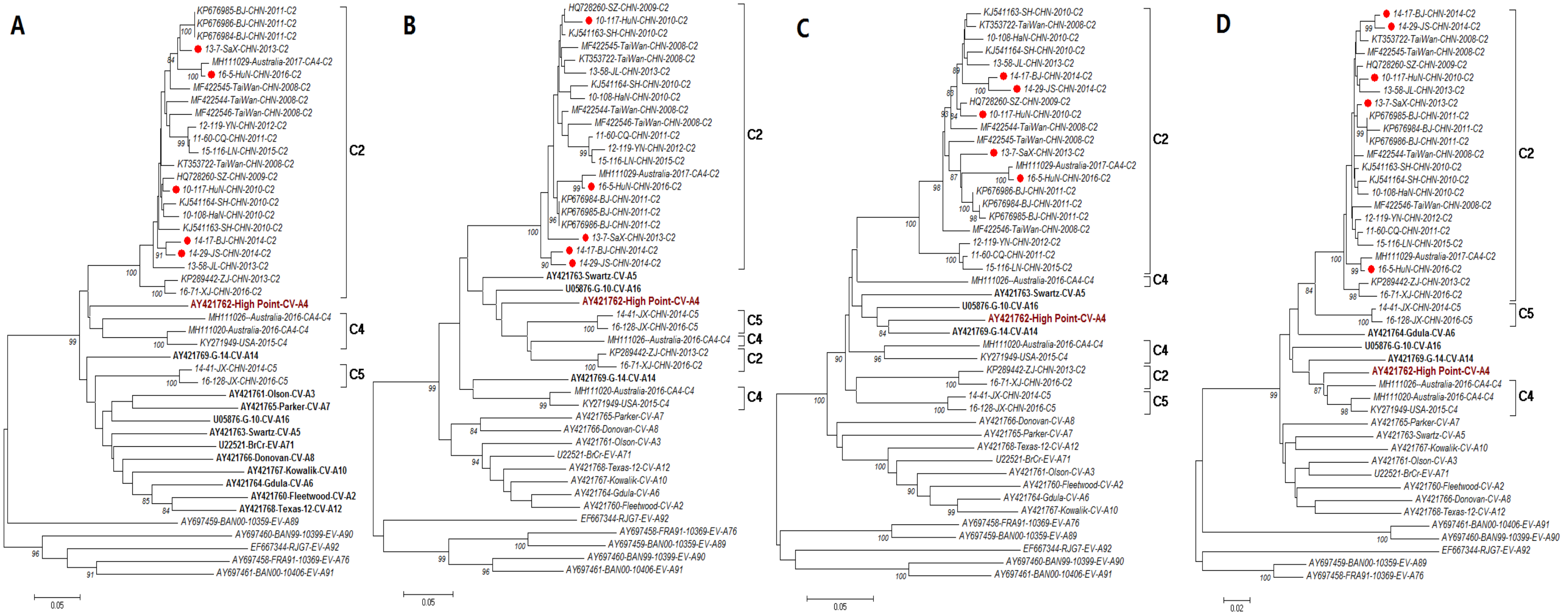

Supplementary Figures S4: Phylogenetic dendrograms showing the relationships amongst EV-A isolates using the different genomic regions. The neighbor-joining trees were constructed from alignment of the2A (A), 2B (B), 2C (C), 5’UTR (D), respectively. Solid circle indicated strains isolates of severe cases in this study.

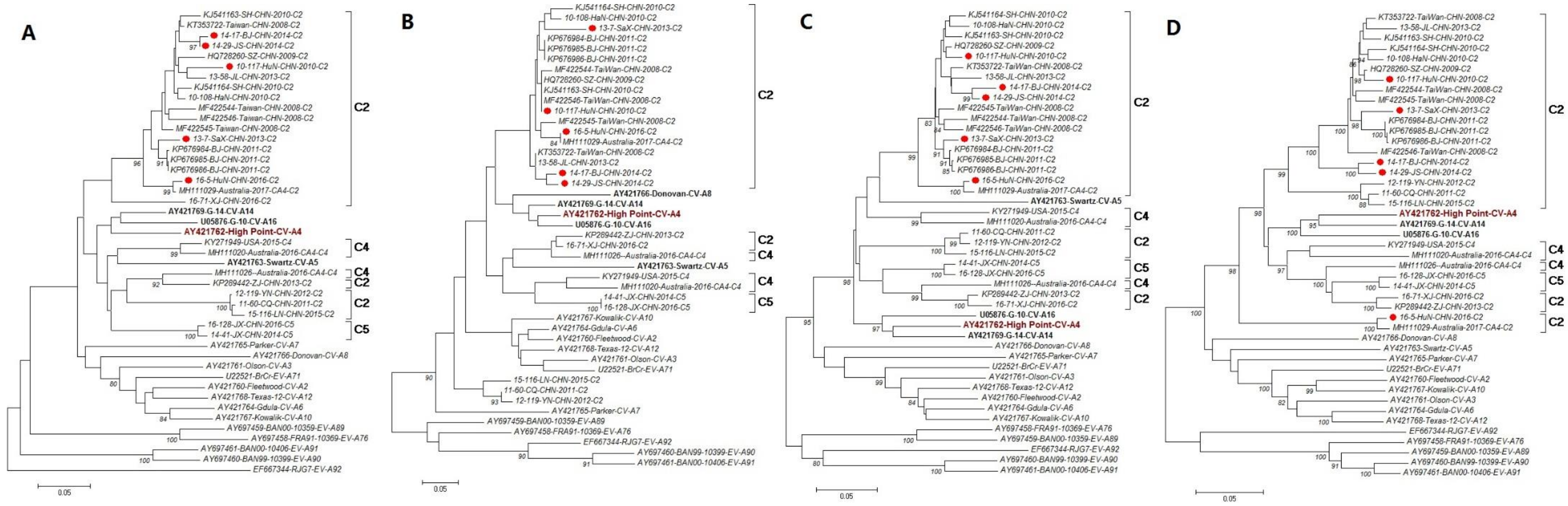

Supplementary Figures S5: Phylogenetic dendrograms showing the relationships amongst EV-A isolates using the different genomic regions. The neighbor-joining trees were constructed from alignment of the 3A (A), 3B (B), 3C (C), 3D (D), respectively. Hollow circle indicated strains of mild cases in this study, rhombus indicated strains isolates of severe cases in this study.

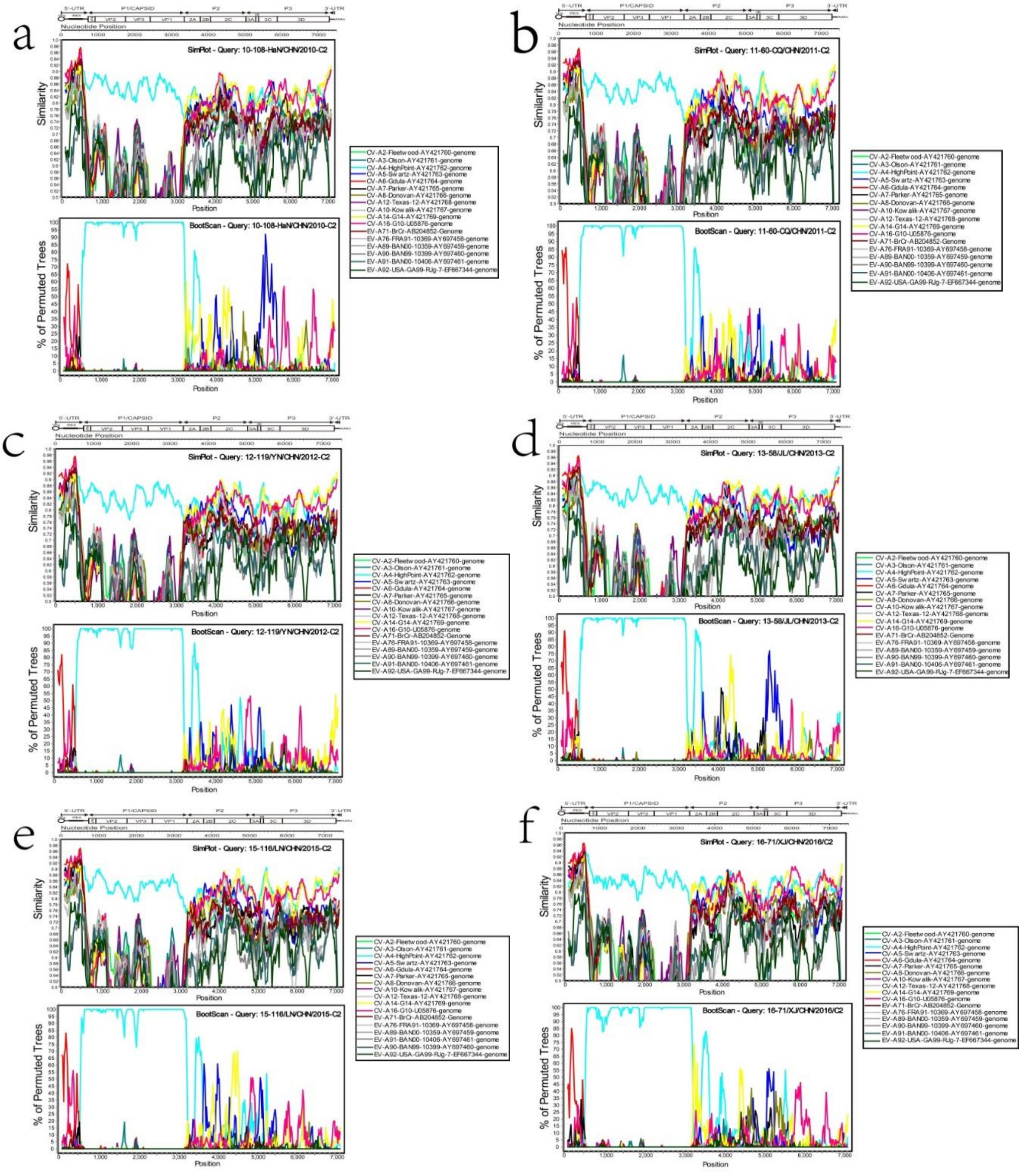

Supplementary Figures S6: Similarity plot and bootscan analysis of C2 sub-genotype of CV-A4 isolated from HFMD mild cases in mainland China with the EV-A prototype strains. A sliding window of 500 nucleotides moving in 20 nucleotides steps was used in this analysis. (a)10-108-HaN-CHN-2010; (b)11-60-CQ-CHN-2011; (c)12-119-YN-CHN-2012; (d)13-58-JL-CHN-2013; (e)15-116-LN-CHN-2015; (f)16-71-XJ-CHN-2016.

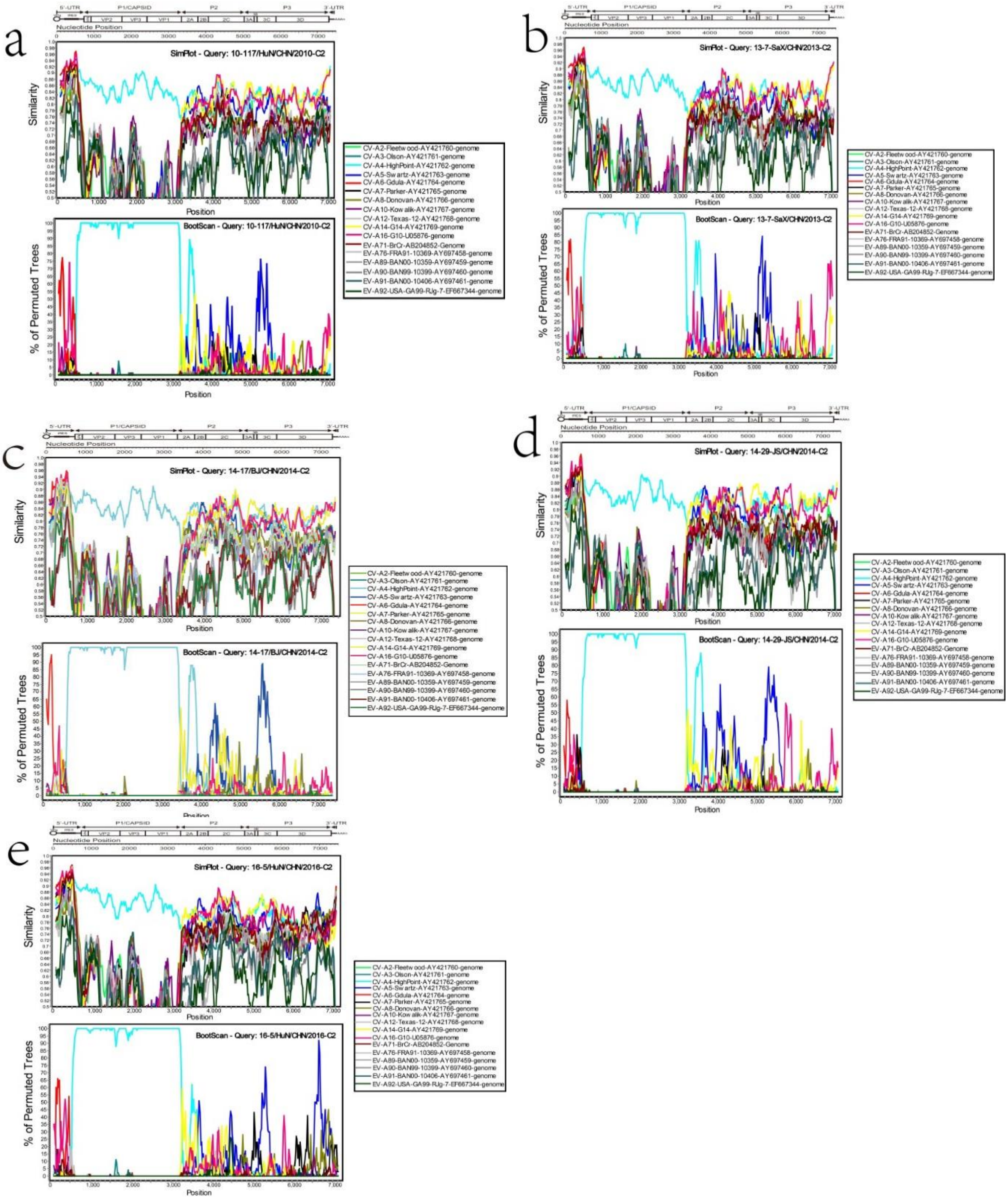

Supplementary Figures S7. Similarity plot and bootscanning analysis of C2 sub-genotype of CV-A4 isolated from HFMD severe cases in mainland China with the EV-A prototype strains. A sliding window of 500 nucleotides moving in 20 nucleotides steps was used in this analysis. (a)10-117-HuN-CHN-2010; (b)13-7-SaX-CHN-2013; (c)14-17-BJ-CHN-2014; (d)14-29-JS-CHN-2014; (e)16-5-HuN-CHN-2016.
