# Supplementary material for: Emerging recombination of the C2 sub-genotype of HFMD-associated CV-A4 is persistently and extensively circulating in China
Source: Sci Rep. 2019 Sep 20;9:13668. doi: 10.1038/s41598-019-49859-7 (PMC6754396; doi:10.1038/s41598-019-49859-7)
Supplement: Supplementary file 2 — supplemental tables [file 41598_2019_49859_MOESM2_ESM.pdf]

Emerging recombination of the C2 sub-genotype of HFMD-associated CV-A4 is persistently and extensively circulating in China

Tianjiao Ji[1], Yue Guo[1], Likun Lv[2], Jianxing Wang[3], Yong Shi[4], Qiuli Yu[5], Fan Zhang[6], Wenbin Tong[7], Jiangtao Ma[8], Hanri Zeng[9], Hua Zhao[10], Yong Zhang[1],Taoli Han[1], Yang Song[1], Dongmei Yan[1], Qian Yang[1], Shuangli Zhu[1], Yan Zhang[1]* and Wenbo Xu[1]*

Table S1-1. Temporal and serotype distribution of EV isolates circulating in China from 2008 to 2017

Year	Number of isolates	EV-A71	CV-A16	Other EV			
				Total	CV-A6	CV-A10	CV-A4
2008	389	236	94	59	1	0	1
2009	601	399	130	72	4	11	0
2010	1644	1026	475	143	17	38	17
2011	1204	805	285	114	10	17	
2012	1288	625	518	145	9	68	13
2013	2490	1022	541	927	488	84	15
2014	2618	1203	1006	409	93	170	44
2015	2598	1069	850	679	352	127	61
2016	2525	975	768	782	330	340	81
2017	2881	1289	710	882	562	147	61
Total	18238	8649	5377	4212	1866	1002	288

Table S1-2. Geographic distribution of 288 Chinese CV-A4 strains from 2008 to 2017

Year	North China	East China	Central China	South China	Southwest China	Northwest China	Northeast China	Total
2008	0	1	0	0	0	0	0	1
2009	0	0	0	0	0	0	0	0
2010	3	0	6	6	0	2	0	17
2011	4	0	0	0	2	2	0	8
2012	5	0	3	1	4	0	0	13
2013	3	1	7	0	1	2	1	15
2014	2	10	3	8	7	10	4	44
2015	27	20	1	0	8	3	2	61
2016	19	22	5	4	7	6	5	68
2017	33	3	7	2	10	5	1	61
Total	96	57	32	21	39	30	13	288

Note:
North China: Tianjin, Beijing, Hebei, Shanxi
East China: Fujian, Anhui, Jiangsu, Zhejiang, Shanghai, Shandong, Jiangxi
Central China: Henan, Hubei, Hunan
South China: Guangdong, Hainan
Southwest China: Sichuan, Chongqing, Yunnan
Northwest China: Shaanxi, Ningxia, Qinghai, Gansu, Xinjiang
Northeast China: Liaoning, Jilin, Heilongjiang

Table S2. List of the 142 complete VP1 sequences of CV-A4 strains selected to generate phylogenetic dendrograms.

Strains name	Country	Provinces	Isolated years	Source of specimen	Disease Type	GenBank accession No.	Origin
17-238-HeB-CHN-2017	CHN	HeB	2017	HFMD	severe case	MK388444	This study
10-12-HeB-CHN-2010	CHN	HeB	2010	HFMD	mild case	MK388445	This study
10-40-NX-CHN-2010	CHN	NX	2010	HFMD	mild case	MK388446	This study
11-114-NX-CHN-2011	CHN	NX	2011	HFMD	mild case	MK388447	This study
12-5-GD-CHN-2012	CHN	GD	2012	HFMD	mild case	MK388448	This study
12-55-HuN-CHN-2012	CHN	HuN	2012	HFMD	severe case	MK388449	This study
12-38-HeB-CHN-2012	CHN	HeB	2012	HFMD	mild case	MK388450	This study
13-11-HeN-CHN-2013	CHN	HeN	2013	HFMD	severe case	MK388451	This study
13-4-HeB-CHN-2013	CHN	HeB	2013	HFMD	mild case	MK388452	This study
13-49-BJ-CHN-2013	CHN	BJ	2013	HFMD	mild case	MK388453	This study
13-66-CQ-CHN-2013	CHN	CQ	2013	HFMD	mild case	MK388454	This study
13-89-HeN-CHN-2013	CHN	HeN	2013	HFMD	mild case	MK388455	This study
13-95-SD-CHN-2013	CHN	SD	2013	HFMD	mild case	MK388456	This study
14-6-QH-CHN-2014	CHN	QH	2014	HFMD	mild case	MK388457	This study
14-28-CQ-CHN-2014	CHN	CQ	2014	HFMD	mild case	MK388458	This study
14-40-QH-CHN-2014	CHN	QH	2014	HFMD	mild case	MK388459	This study
14-166-XJ-CHN-2014	CHN	XJ	2014	HFMD	mild case	MK388460	This study
14-6-SC-CHN-2014	CHN	SC	2014	HFMD	mild case	MK388461	This study
14-107-HuN-CHN-2014	CHN	HuN	2014	HFMD	mild case	MK388462	This study
14-66-HuB-CHN-2014	CHN	HuB	2014	HFMD	mild case	MK388463	This study
14-23-JX-CHN-2014	CHN	JX	2014	HFMD	mild case	MK388464	This study
14-10-YN-CHN-2014	CHN	YN	2014	HFMD	mild case	MK388465	This study
14-21-SD-CHN-2014	CHN	SD	2014	HFMD	mild case	MK388466	This study
14-28-GD-CHN-2014	CHN	GD	2014	HFMD	mild case	MK388467	This study
14-49-GS-CHN-2014	CHN	GS	2014	HFMD	/	MK388468	This study
14-89-SaX-CHN-2014	CHN	SaX	2014	HFMD	mild case	MK388469	This study
14-199-LN-CHN-2014	CHN	LN	2014	HFMD	mild case	MK388470	This study
15-99-GS-CHN-2015	CHN	GS	2015	HFMD	severe case	MK388471	This study
15-20-SC-CHN-2015	CHN	SC	2015	HFMD	mild case	MK388472	This study
15-56-SD-CHN-2015	CHN	SD	2015	HFMD	mild case	MK388473	This study
15-89-NX-CHN-2015	CHN	NX	2015	HFMD	mild case	MK388474	This study
15-175-HeN-CHN-2015	CHN	HeN	2015	HFMD	mild case	MK388475	This study
15-86-TJ-CHN-2015	CHN	TJ	2015	HFMD	mild case	MK388476	This study
15-134-HeB-CHN-2015	CHN	HeB	2015	HFMD	mild case	MK388477	This study
15-80-XJ-CHN-2015	CHN	XJ	2015	HFMD	mild case	MK388478	This study
16-60-CQ-CHN-2016	CHN	CQ	2016	HFMD	mild case	MK388479	This study
16-139-HeB-CHN-2016	CHN	HeB	2016	HFMD	severe case	MK388480	This study
16-102-SaX-CHN-2016	CHN	SaX	2016	HFMD	severe case	MK388481	This study
16-35-HuN-CHN-2016	CHN	HuN	2016	HFMD	severe case	MK388482	This study
16-121-JS-CHN-2016	CHN	JS	2016	HFMD	severe case	MK388483	This study
16-69-TJ-CHN-2016	CHN	TJ	2016	HFMD	mild case	MK388484	This study
16-43-TJ-CHN-2016	CHN	TJ	2016	HFMD	mild case	MK388485	This study
16-17-AN-CHN-2016	CHN	AN	2016	HFMD	mild case	MK388486	This study
16-23-AN-CHN-2016	CHN	AN	2016	HFMD	mild case	MK388487	This study
16-62-SC-CHN-2016	CHN	SC	2016	HFMD	mild case	MK388488	This study

16-112-HLJ-CHN-2016	CHN	HLJ	2016	HFMD	mild case	MK388489	This study
16-79-NX-CHN-2016	CHN	NX	2016	HFMD	mild case	MK388490	This study
16-37-JX-CHN-2016	CHN	JX	2016	HFMD	mild case	MK388491	This study
16-19-GD-CHN-2016	CHN	GD	2016	HFMD	mild case	MK388492	This study
17-25-HaN-CHN-2017	CHN	HaN	2017	HFMD	/	MK388493	This study
17-26-NX-CHN-2017	CHN	NX	2017	HFMD	mild case	MK388494	This study
17-62-ZJ-CHN-2017	CHN	ZJ	2017	HFMD	mild case	MK388495	This study
17-420-GS-CHN-2017	CHN	GS	2017	HFMD	mild case	MK388496	This study
17-51-JX-CHN-2017	CHN	JX	2017	HFMD	mild case	MK388497	This study
17-79-CQ-CHN-2017	CHN	CQ	2017	HFMD	mild case	MK388498	This study
17-12-LN-CHN-2017	CHN	LN	2017	HFMD	mild case	MK388499	This study
17-107-SX-CHN-2017	CHN	SX	2017	HFMD	mild case	MK388500	This study
17-96-HeN-CHN-2017	CHN	HeN	2017	HFMD	mild case	MK388501	This study
17-305-HeB-CHN-2017	CHN	HeB	2017	HFMD	mild case	MK388502	This study
17-76-TJ-CHN-2017	CHN	TJ	2017	HFMD	mild case	MK388503	This study
14-90-SD-CHN-2014	CHN	SD	2014	HFMD	mild case	MK388504	This study
10-108-HaN-CHN-2010	CHN	HaN	2010	HFMD	mild case	MK391063	This study
10-117-HuN-CHN-2010	CHN	HuN	2010	HFMD	mild case	MK391064	This study
11-60-CQ-CHN-2011	CHN	CQ	2011	HFMD	mild case	MK391065	This study
12-119-YN-CHN-2012	CHN	YN	2012	HFMD	mild case	MK391066	This study
13-7-SaX-CHN-2013	CHN	SaX	2013	HFMD	severe case	MK391067	This study
13-58-JL-CHN-2013	CHN	JL	2013	HFMD	mild case	MK391068	This study
14-17-BJ-CHN-2014	CHN	BJ	2014	HFMD	severe case	MK391069	This study
14-29-JS-CHN-2014	CHN	JS	2014	HFMD	severe case	MK391070	This study
14-41-JX-CHN-2014	CHN	JX	2014	HFMD	mild case	MK391071	This study
15-116-LN-CHN-2015	CHN	LN	2015	HFMD	mild case	MK391072	This study
16-5-HuN-CHN-2016	CHN	HuN	2016	HFMD	severe case	MK391073	This study
16-71-XJ-CHN-2016	CHN	XJ	2016	HFMD	mild case	MK391074	This study
16-128-JX-CHN-2016	CHN	JX	2016	HFMD	mild case	MK391075	This study
KJ818314-HuN-CHN-2010	CHN	HuN	2010	HFMD	severe case	KJ818314	GenBank
KJ818315-HuN-CHN-2010	CHN	HuN	2010	HFMD	severe case	KJ818315	GenBank
KJ818317-HuN-CHN-2010	CHN	HuN	2010	HFMD	severe case	KJ818317	GenBank
KJ818320-HuN-CHN-2012	CHN	HuN	2012	HFMD	severe case	KJ818320	GenBank
KY978560-SD-CHN-2013	CHN	SD	2013	AFP	/	KY978560	GenBank
KJ818307-CQ-CHN-2011	CHN	CQ	2011	HFMD	mild case	KJ818307	GenBank
KJ818309-CQ-CHN-2012	CHN	CQ	2012	HFMD	mild case	KJ818309	GenBank
LC175758-SC-CHN-2006	CHN	SC	2006	AFP	/	LC175758	GenBank
LC175762-SC-CHN-2007	CHN	SC	2007	AFP	/	LC175762	GenBank
KY978567-SD-CHN-1996	CHN	SD	1996	AFP	/	KY978567	GenBank
LC412060-YN-CHN-2014	CHN	YN	2014	AFP	/	LC412060	GenBank
LC412066-YN-CHN-2014	CHN	YN	2014	AFP	/	LC412066	GenBank
JQ715708-JL-CHN-2006	CHN	JL	2006	AFP	/	JQ715708	GenBank
KY978541-SD-CHN-2003	CHN	SD	2003	AFP	/	KY978541	GenBank
GQ253372-SD-CHN-2008	CHN	SD	2008	AFP	/	GQ253372	GenBank
GQ253375-SD-CHN-2006	CHN	SD	2006	AFP	/	GQ253375	GenBank
HQ728260-GD-CHN-2009	CHN	GD	2009	HFMD	/	HQ728260	GenBank
JQ715709-JL-CHN-2006	CHN	JL	2006	AFP	/	JQ715709	GenBank
JQ715710-JL-CHN-2007	CHN	JL	2007	healthy people	/	JQ715710	GenBank
KC867060-GD-CHN-2008	CHN	GD	2008	HFMD	/	KC867060	GenBank
KC867063-GD-CHN-2012	CHN	GD	2012	HFMD	/	KC867063	GenBank
KF150144-SD-CHN-2010	CHN	SD	2010	AFP	/	KF150144	GenBank
KJ541163-SH-CHN-2010	CHN	SH	2010	HFMD	mild case	KJ541163	GenBank

KJ818305-AH-CHN-2008	CHN	AH	2008	HFMD	mild case	KJ818305	GenBank
KJ818310-GD-CHN-2010	CHN	GD	2010	HFMD	mild case	KJ818310	GenBank
KJ818313-HaN-CHN-2010	CHN	HaN	2010	HFMD	mild case	KJ818313	GenBank
KJ818321-JS-CHN-2012	CHN	JS	2012	HFMD	mild case	KJ818321	GenBank
KJ818325-SaX-CHN-2010	CHN	SaX	2010	HFMD	mild case	KJ818325	GenBank
KP398833-SH-CHN-2010	CHN	SH	2010	HFMD	/	KP398833	GenBank
KP676957-BJ-CHN-2011	CHN	BJ	2011	febrile illness	/	KP676957	GenBank
KX982676-CHN-2016	CHN	/	2016	N/A	/	KX982676	GenBank
KP289442-ZJ-CHN-2013	CHN	ZJ	2013	HFMD	/	KP289442	GenBank
LC169516-SC-CHN-2015	CHN	SC	2015	HFMD	/	LC169516	GenBank
KY978537-SD-CHN-2008	CHN	SD	2008	AFP	/	KY978537	GenBank
KY978539-SD-CHN-2009	CHN	SD	2009	AFP	/	KY978539	GenBank
KY978547-SD-CHN-2010	CHN	SD	2010	AFP	/	KY978547	GenBank
KY978550-SD-CHN-2011	CHN	SD	2011	AFP	/	KY978550	GenBank
KY978559-SD-CHN-2013	CHN	SD	2013	AFP	/	KY978559	GenBank
KY978561-SD-CHN-2014	CHN	SD	2014	AFP	/	KY978561	GenBank
KY978568-SD-CHN-2015	CHN	SD	2015	AFP	/	KY978568	GenBank
KY978570-SD-CHN-2007	CHN	SD	2007	AFP	/	KY978570	GenBank
LC169513-SC-CHN-2014	CHN	SC	2014	HFMD	/	LC169513	GenBank
LC169515-SC-CHN-2015	CHN	SC	2015	HFMD	/	LC169515	GenBank
LC175759-SC-CHN-2006	CHN	SC	2006	AFP	/	LC175759	GenBank
LC175760-SC-CHN-2006	CHN	SC	2006	AFP	/	LC175760	GenBank
LC175761-SC-CHN-2007	CHN	SC	2007	AFP	/	LC175761	GenBank
LC175764-SC-CHN-2008	CHN	SC	2008	AFP	/	LC175764	GenBank
LC175766-SC-CHN-2009	CHN	SC	2009	AFP	/	LC175766	GenBank
LC175768-SC-CHN-2014	CHN	SC	2014	AFP	/	LC175768	GenBank
LC361273-SC-CHN-2016	CHN	SC	2016	healthy children	/	LC361273	GenBank
AY421762-High Point-USA-1948	USA	/	1048	/	/	AY421762	GenBank
AB457644-Japan-2008	JPN	/	2008	/	/	AB457644	GenBank
GQ176232-Kenya-1999	Kenya	/	1999	HIV	/	GQ176232	GenBank
GQ253377-SD-CHN-1998	CHN	SD	1998	AFP	/	GQ253377	GenBank
KT353722-Taiwan-CHN-2008	CHN	TW	2008	/	/	KT353722	GenBank
JN203505-India-2011	IND	/	2011	AFP	/	JN203505	GenBank
JN203507-India-2011	IND	/	2011	AFP	/	JN203507	GenBank
JN203509-India-2011	IND	/	2011	AFP	/	JN203509	GenBank
JN203510-India-2011	IND	/	2011	AFP	/	JN203510	GenBank
JN203513-India-2011	IND	/	2011	AFP	/	JN203513	GenBank
MH111029-Australia-2017	AUS	/	2017	HFMD	/	MH111029	GenBank
MH111028-Australia-2017	AUS	/	2017	HFMD	/	MH111028	GenBank
MH111026-Australia-2016	AUS	/	2016	HFMD	/	MH111026	GenBank
MH111023-Australia-2016	AUS	/	2016	HFMD	/	MH111023	GenBank
MH111020-Australia-2016	AUS	/	2016	HFMD	/	MH111020	GenBank
KY271949-USA-2015	USA	/	2015	/	/	KY271949	GenBank
KR185978-Russion-2013	RUS	Saint-Petersburg	2013	enterovirus infection	/	KR185978	GenBank
KR185979-Russion-2014	RUS	Saint-Petersburg	2014	enterovirus infection	/	KR185979	GenBank

The names of countries are abbreviated as CHN, China; IND, India; JPN, Japan; USA, United States; AUS, Australia; and RUS, Russia. The provinces in China are abbreviated as BJ, Beijing; JX, Jiangxi; XJ, Xinjiang; SD, Shandong; AH, Anhui; HLJ, Heilongjiang; ZJ, Zhejiang; JS, Jiangsu; SC, Sichuan; GS, Gansu; CQ, Chongqing; HeB, Hebei; HeN, Henan; HuN, Hunan; HaN, Hainan; HuB, Hubei; JL, Jilin; LN, Liaoning; SX, Shanxi; Sax, Shaanxi; NX, Ningxia; GD, Guangdong; SH, Shanghai; TJ, Tianjin; and YN, Yunnan.

Table S3. List of the 29 complete genome sequences of CV-A4 strains selected to generate phylogenetic dendrograms.

Strains name	Country	Provinces	Isolated years	Source of specimen	Disease Type	GenBank accession No.	Origin
10-108-HaN-CHN-2010	CHN	HaN	2010	HFMD	mild case	MK388504	This study
10-117-HuN-CHN-2010	CHN	HuN	2010	HFMD	mild case	MK391063	This study
11-60-CQ-CHN-2011	CHN	CQ	2011	HFMD	mild case	MK391064	This study
12-119-YN-CHN-2012	CHN	YN	2012	HFMD	mild case	MK391065	This study
13-7-SaX-CHN-2013	CHN	SaX	2013	HFMD	severe case	MK391066	This study
13-58-JL-CHN-2013	CHN	JL	2013	HFMD	mild case	MK391067	This study
14-17-BJ-CHN-2014	CHN	BJ	2014	HFMD	severe case	MK391068	This study
14-29-JS-CHN-2014	CHN	JS	2014	HFMD	severe case	MK391069	This study
14-41-JX-CHN-2014	CHN	JX	2014	HFMD	mild case	MK391070	This study
15-116-LN-CHN-2015	CHN	LN	2015	HFMD	mild case	MK391071	This study
16-5-HuN-CHN-2016	CHN	HuN	2016	HFMD	severe case	MK391072	This study
16-71-XJ-CHN-2016	CHN	XJ	2016	HFMD	mild case	MK391073	This study
16-128-JX-CHN-2016	CHN	JX	2016	HFMD	mild case	MK391074	This study
CVA4/SZ/CHN/09	CHN	GD	2009	HFMD	/	MK391075	GenBank
701/SH/CHN/2010	CHN	SH	2010	HFMD	/	KJ541163	GenBank
1047/SH/CHN/2010	CHN	SH	2010	HFMD	/	KJ541164	GenBank
FT/CHN/05	CHN	BJ	2011	febrile illness	/	KP676984	GenBank
FT/CHN/07	CHN	BJ	2011	febrile illness	/	KP676985	GenBank
FT/CHN/27	CHN	BJ	2011	febrile illness	/	KP676986	GenBank
P1033/China/2013	CHN	ZJ		HFMD	/	KP289442	GenBank
KY271949-USA-2015	USA	/	2015	N/A	/	KY271949	GenBank
KT353722-Taiwan-CHN-2008	CHN	TW	2008	N/A	/	KT353722	GenBank
MF422544-Taiwan-CHN-2008	CHN	TW	2008	N/A	/	MF422544	GenBank
MF422545-Taiwan-CHN-2008	CHN	TW	2008	N/A	/	MF422545	GenBank
MF422546-Taiwan-CHN-2008	CHN	TW	2008	N/A	/	MF422546	GenBank
AY421762-High Point-USA-1948	USA	/	1948	N/A	/	AY421762	GenBank
MH111020-Australia-2016	AUS	/	2016	HFMD	/	MH111020	GenBank
MH111026-Australia-2016	AUS	/	2016	HFMD	/	MH111026	GenBank
MH111029-Australia-2017	AUS	/	2017	HFMD	/	MH111029	GenBank

The names of countries are abbreviated as CHN, China; USA, United States; and AUS, Australia. The provinces in China are abbreviated as BJ, Beijing; JX, Jiangxi; XJ, Xinjiang; JS, Jiangsu; CQ, Chongqing; HuN, Hunan; JL, Jilin; LN, Liaoning; GD, Guangdong; TJ, Tianjin; YN, Yunnan and TW, Taiwan.

Table S4. Pairwise nucleotide sequences identities between 29 genomic sequences of CV-A4 and prototype strains of EV-A species

Region	Identity (%)																
	CV-A2	CV-A3	CV-A4	CV-A5	CV-A6	CV-A7	CV-A8	CV-A10	CV-A12	CV-A14	CV-A16	EV-A71	EV-A76	EV-A89	EV-A90	EV-A91	EV-A92
Genome	71.5-74.7	72.4-75.3	82.2-85.1	72.6-75.5	72.3-75.3	71.4-74.0	72.3-75.2	71.9-74.6	71.2-74.2	73.7-76.5	73.8-76.8	71.1-73.9	67.8-69.9	68.3-70.1	67.0-69.0	67.1-69.2	64.7-67.5
5'-UTR	71.1-83.5	72.6-85.5	76.0-89.6	72.3-84.4	75.6-88.8	72.3-85.2	71.0-82.9	71.5-84.1	71.1-83.5	71.3-85.4	74.4-87.6	72.7-85.0	66.8-79.7	65.8-78.6	61.4-72.4	60.8-72.9	58.8-70.6
P1	65.9-66.7	66.3-67.4	84.0-86.1	63.4-64.7	65.6-66.6	62.1-63.3	65.7-66.9	66.2-67.6	63.7-64.4	59.8-60.8	61.1-62.2	61.1-62.2	63.1-64.3	63.6-64.9	63.1-63.7	63.1-64.2	61.8-62.9
VP4	79.7-82.6	73.9-75.3	95.6-100.0	65.2-69.5	71.0-75.3	78.2-81.1	73.9-76.8	72.4-75.3	75.3-78.2	69.5-69.9	73.9-75.3	66.6-68.1	72.4-76.8	72.4-76.8	72.4-75.3	72.4-76.8	72.4-76.8
VP2	74.5-79.2	76.8-81.1	94.1-99.2	70.1-75.6	78.0-81.9	69.8-74.1	74.9-79.2	75.6-80.3	70.5-75.2	61.9-66.2	69.8-74.5	67.0-72.5	71.4-76.8	72.5-76.8	72.1-77.2	70.2-75.6	66.2-71.7
VP3	70.2-72.3	70.1-72.5	83.0-87.5	65.6-67.4	67.7-70.8	65.5-68.0	69.4-71.3	69.7-71.9	66.7-67.5	62.6-64.0	61.9-63.8	64.3-66.2	65.8-68.1	66.8-67.8	64.7-67.7	65.9-68.6	65.0-67.9
VP1	57.9-60.5	59.4-61.3	84.4-85.6	57.0-59.8	58.0-59.7	52.8-55.0	59.4-61.1	60.2-62.8	55.9-57.8	51.1-53.1	55.8-56.9	54.4-55.5	55.1-56.8	55.8-58.6	56.2-57.8	55.5-57.6	53.9-55.3
P2	76.4-78.4	77.4-79.8	81.6-83.5	80.5-82.7	77.5-79.5	78.0-79.4	78.2-80.2	76.3-79.4	78.3-79.7	82.8-84.3	82.1-83.6	78.6-79.5	71.0-72.6	72.5-73.8	69.9-72.2	71.4-73.0	69.1-71.6
2A	73.7-79.5	76.0-78.2	77.7-82.8	77.5-80.0	76.4-79.5	75.5-77.3	76.8-79.7	74.6-78.6	75.7-80.4	80.0-84.0	78.0-81.3	77.1-80.4	65.5-68.6	67.7-71.7	65.5-67.7	65.5-69.5	63.7-66.8
2B	74.4-78.7	75.4-79.4	78.7-84.8	78.7-85.1	74.7-78.7	74.7-78.7	74.4-79.4	76.4-79.1	76.0-79.1	79.1-84.5	80.8-84.8	76.4-80.1	65.6-69.0	65.9-68.3	66.6-70.3	68.0-72.0	68.0-72.0
2C	77.0-79.8	78.1-81.0	81.5-85.2	81.1-83.5	77.3-81.2	79.1-81.2	79.8-81.2	76.5-80.9	79.2-81.6	83.5-86.4	82.9-85.5	78.7-80.9	74.8-76.6	74.9-77.3	72.1-75.3	73.6-75.5	71.7-74.8
P3	76.4-79.6	76.8-79.6	81.9-84.9	78.0-80.2	76.9-79.5	75.8-79.7	77.2-79.4	77.0-78.7	77.1-79.5	83.0-89.5	81.0-87.7	76.9-79.5	71.3-73.5	71.5-73.6	71.2-73.7	71.8-74.6	70.2-72.9
3A	72.4-82.1	71.3-82.9	77.1-84.8	72.4-83.7	72.0-81.3	73.2-81.3	70.9-78.2	74.0-80.2	70.9-81.3	77.9-86.4	76.7-86.0	71.7-80.6	68.6-73.2	67.0-75.1	69.3-75.5	70.1-76.7	64.7-68.9
3B	68.1-81.8	69.6-78.7	78.7-87.8	74.2-81.8	71.2-81.8	65.1-77.2	72.7-81.8	72.7-81.8	69.6-83.3	77.2-90.0	80.3-86.3	69.6-77.2	66.6-75.7	68.1-78.7	56.0-63.6	56.0-63.6	59.0-71.2
3C	75.9-79.7	76.1-78.8	81.6-86.7	78.8-83.9	76.5-78.3	75.2-77.5	75.5-79.2	75.0-78.3	77.7-80.5	80.5-85.6	80.5-84.3	76.3-78.3	71.4-75.5	73.0-75.4	73.0-74.8	72.3-75.5	70.1-73.4
3D	74.9-80.0	75.6-80.0	80.7-84.9	76.0-79.6	75.6-80.0	75.3-80.5	76.9-79.9	75.3-78.8	75.8-79.3	79.3-85.2	80.3-84.9	75.9-80.2	70.2-74.0	70.2-74.1	70.4-74.3	71.5-74.7	70.4-73.6

Table S5. List of nucleotide sequences of primer for amplification of the whole genome sequences of CV-A4

Primer	Nucleotide position (nt)	Primer sequence（5′- 3′）	Orientation	Reference
F1	1-21	TTTAAAACAGCCTGTGGGTTG	Forward	[1]
R1-G	316-335	CTCATCGACCTGATCTACAC	Reverse	This study
cox243	243-266	ACCCGGCTAACTACTTCGAGAAAC	Forward	[1]
cox1281-G	1281-1300	TTACTGGCATTACATTGCAC	Reverse	This study
fcox1108-G	1108-1127	AACCCACCAGACCTGATGTT	Forward	This study
fcox2093	2093-2116	ATGAATGAGCCCGTAAACATGAAA	Reverse	[1]
M1908	1908-1930	AGGTGTAAGCCGGTTGCTCATAC	Forward	[1]
M2932	2932-2954	CACTGGAACGATTCTCGAGCATC	Reverse	[1]
CV-A4-VP1-F	2341-2360	ACACGCCGAACGAAGCTAAT	Forward	This study
CV-A4-VP1-R	3433-3452	TTATGTGTGGCTAGATGGCG	Reverse	This study
N2846	2846-2866	TCACCTTCGTCACCAATCTAG	Forward	[1]
N3857	3857-3876	CCTGGAGACTGCGTCAGTGA	Reverse	[1]
bcox3615	3615-3642	CAGTGAGTACTACCCTGCCAGGTATCAA	Forward	[1]
bcox4932	4932-4958	TGCGGTGTTATTTTCAGAGCACAGTTTG	Reverse	[1]
ccox4610	4610-4635	AGCAAGTGGTCACTGTCATGGATGA	Forward	[1]
ccox5933	5933-5958	CCTGCACAAAAGCCCTGCCTGCCAT	Reverse	[1]
CV-A4-5321F	5321-5340	GTATGTCATCTACAAGCTCT	Forward	This study
CV-A4-6601F	6601-6620	AACACATCTGGGTTGCACCC	Reverse	This study
dcox5856	5856-5881	TGGAGGAGTAGTTACATCAGTTGGA	Forward	[1]
dcox7403	7403-7428	CAGTTATGTTCACGACCAGATTTCT	Reverse	[1]
ecox-a(race pcr 3')	7147-7173	ATGCCAATGAAGGAGATTCATGAGTCC	Forward	[1]

[1] Li Jin-Song, Dong Xiao-Gen, Qin Menget al. Outbreak of febrile illness caused by coxsackievirus A4 in a nursery school in Beijing, China[J]. Virology Journal, 2015, 12（1）: 92.
